# Predicting the Impact of COVID-19 and the Potential Impact of the Public Health Response on Disease Burden in Uganda

**DOI:** 10.4269/ajtmh.20-0546

**Published:** 2020-07-23

**Authors:** David Bell, Kristian Schultz Hansen, Agnes N. Kiragga, Andrew Kambugu, John Kissa, Anthony K. Mbonye

**Affiliations:** 1 Independent Consultant, Issaquah, Washington;; 2 Department of Public Health, Centre for Health Economics and Policy, University of Copenhagen, Copenhagen, Denmark;; 3 Infectious Diseases Institute, School of Medicine, College of Health Sciences, Makerere University, Kampala, Uganda;; 4 Uganda Ministry of Health, Division of Health Information, Kampala, Uganda;; 5 School of Public Health, College of Health Sciences, Makerere University, Kampala, Uganda

## Abstract

The COVID-19 pandemic and public health “lockdown” responses in sub-Saharan Africa, including Uganda, are now widely reported. Although the impact of COVID-19 on African populations has been relatively light, it is feared that redirecting focus and prioritization of health systems to fight COVID-19 may have an impact on access to non–COVID-19 diseases. We applied age-based COVID-19 mortality data from China to the population structures of Uganda and non-African countries with previously established outbreaks, comparing theoretical mortality and disability-adjusted life years (DALYs) lost. We then predicted the impact of possible scenarios of the COVID-19 public health response on morbidity and mortality for HIV/AIDS, malaria, and maternal health in Uganda. Based on population age structure alone, Uganda is predicted to have a relatively low COVID-19 burden compared with an equivalent transmission in comparison countries, with 12% of the mortality and 19% of the lost DALYs predicted for an equivalent transmission in Italy. By contrast, scenarios of the impact of the public health response on malaria and HIV/AIDS predict additional disease burdens outweighing that predicted from extensive SARS-CoV-2 transmission. Emerging disease data from Uganda suggest that such deterioration may already be occurring. The results predict a relatively low COVID-19 impact on Uganda associated with its young population, with a high risk of negative impact on non–COVID-19 disease burden from a prolonged lockdown response. This may reverse hard-won gains in addressing fundamental vulnerabilities in women and children’s health, and underlines the importance of tailoring COVID-19 responses according to population structure and local disease vulnerabilities.

## INTRODUCTION

The COVID-19 pandemic is redirecting focus and prioritization of health systems globally. Public health responses have been dominated by enforced social distancing and stay-at-home interventions, characterized as “lockdowns,” advocated by the World Health Organization (WHO).^1^ As reported SARS-CoV-2 infections increased on the African continent, countries implemented measures previously used elsewhere including closure of workplaces and severe restrictions on travel, aimed at reducing transmission and subsequent pressure on intensive healthcare facilities.^2^ Reducing close contact between people should reduce the basic reproduction rate.^3^

Relatively few cases of COVID-19 have been recorded to date in sub-Saharan African countries.^4^ However, sub-Saharan Africa is considered particularly vulnerable to COVID-19 because of the relatively weak health service infrastructure and low clinician–population ratios,^5^ relatively limited laboratory capacity,^6^ and a higher rate of underlying conditions including malnutrition and anemia, HIV/AIDs, and chronic respiratory conditions due to tuberculosis and air pollution.^7^ There is paucity of data on interactions between SARS-CoV-2 infection and other acute febrile disease, such as malaria.^8^ Although these conditions could exacerbate or ameliorate COVID-19, they increase the population’s dependence on health service access and the commodity supply lines that support it, including access to long-term medication and care in acute illness. Access to health services is also essential for reducing poor antenatal and birthing outcomes.

Health services are at risk in the COVID-19 outbreak through various mechanisms. Clinicians and direct care givers of COVID-19 patients have a disproportionately higher mortality than the general age-adjusted population.^9^ Closure of logistics-related workplaces and transport services interrupts supply lines.^10^ Travel bans and reduction in public transportation limit access, and public perceptions of increased risk of SARS-CoV-2 infection near health facilities dissuade attendance, whereas clinic activities considered “non-urgent,” such as antenatal care, may be postponed.^11^

In contradistinction to potential health vulnerabilities, sub-Saharan African countries could be “protected” from COVID-19 mortality by an age structure differing significantly from countries where mortality has been particularly high—such as Italy, Spain, the United States, and in Hubei Province in China.^12,13^ Countries with far higher ratios of children to older adults are expected to have lower overall mortality and higher rates of asymptomatic infection.^14^ Age comparisons are of particular importance to public health, as disability-adjusted life years (DALYs), fundamental to assessing relative burden, are highly influenced by age of death or onset of long-term disability.^15^

Uganda typifies many of the characteristics of sub-Saharan countries. With a fertility rate greater than 5, 50% of the population are estimated to be younger than 17 years, and only 1.6% are ≥ 70 years in 2020.^12,16^ Total incidence and mortality from malaria (12,356,577, 13,203) and tuberculosis (86,000 and 19,000, respectively) in 2018 imparted a significant burden on its 41 million people.^17,18^ Maternal mortality ratio was 368 per 100,000 live births in 2016,^19^ whereas 100,000 HIV-positive women were dependent on antiretroviral therapy (ART) to prevent mother-to-child transmission in 2018.^20^ Success of the HIV/AIDS program has reduced AIDS-related mortality from 43,000 in 1990 to 23,500 in 2018,^20^ resulting in a growing number of people living with HIV (1,400,000 in 2018) and dependent on frequent monitoring, ART, and treatment of coinfections.^20^ With low ratios of physicians to population (0.1/1,000 people) and hospital beds (0.5/1,000),^21^ Uganda has a relatively fragile health system with limited capacity to expand critical care services.

In view of this, we hypothesize that mortality in Uganda accrued from COVID-19 and from interruptions to other disease programs resulting from the response will differ greatly from those of outbreak countries in Europe, North America, and East Asia. As of June 22, 2020, Uganda had conducted 166,917 tests for COVID-19 and of these, 770 (0.5%) tested positive, whereas 492 persons have recovered, and no deaths have been registered.^22^ The neighboring country of Kenya has reported higher community transmission with five-hold higher numbers of COVID-19 cases.^23^ A significant proportion of the Uganda cases are among transborder cargo truck drivers, underlining the threat of continued cross-border introduction.^22^

We therefore predicted the potential burden of COVID-19 in Uganda based on its population age structure in comparison to an equivalent outbreak in other countries with established COVID-19 transmission. We calculated mortality and DALYs lost, a summary measure of lifetime impact combining time lost through premature death and time lived in states of less than optimal health.^15^ We then predicted the potential for impact of a COVID-19 lockdown response on the burden of selected diseases in Uganda, where restrictions on travel and workplace attendance have been implemented by the government of Uganda since March 12, 2020. We grounded this against limited publicly available data on changes in health burden in Uganda over recent months.

## METHODS

Data collection was conducted in Uganda, which has a sporadic transmission of COVID-19 and conditions generalizable across several sub-Saharan countries.^4^

### Data sources to assess recent trends in disease burden.

Information on the Ugandan population structure was obtained from the Uganda Bureau of Statistics census report, whereas data on comparison countries were obtained from UN data (Supplemental Table S1).^13,24^

Data on HIV and tuberculosis were obtained from the aggregated President’s Emergency Plan for Aids Relief (PEPFAR) weekly surge reports generated from data across 13 PEPFAR Uganda implementing partners,^25^ including the persons newly diagnosed with HIV as per the Uganda national HIV/AIDS treatment guidelines.^26^ Additional data on age-stratified HIV prevalence were obtained from the Uganda Population-based HIV Impact Assessment final report.^27^

Age-stratified malaria incidence for 2019–2020 was obtained from the Uganda Health Management Information System (HMIS) quarterly reporting.^28^ Maternal mortality data including deliveries from January 2019 to March 2020 inclusive were similarly obtained from the Uganda HMIS quarterly reporting.^28^

### Calculation of the number of DALYs lost.

Calculations of DALYs lost followed broadly the methods outlined for the most recent WHO global burden of disease estimates.^29^ This involved defining life years lost by age as the difference between actual age of death and the life expectancy in a standard life table reflecting the highest life expectancy in the world today and a set of disability weights to reflect the relative severity of diseases. The unequal age-weighting function and discounting of future life years applied in earlier DALY versions were excluded.^15^

COVID-19 disease burden and excess HIV, malaria, and maternal mortality were calculated by multiplying the DALYs lost for a single health event by the population incidence and deaths by age-group. Disability-adjusted life years for COVID-19 are based on age-related mortality reported by Verity et al. from the China outbreak, applied to age structures of comparison countries with relatively high COVID-19 burden (the United States, China, Italy, and Spain) and Iceland,^30^ where testing rates have been relatively high.^23^ For the sake of comparison, a 20% total detectable infection rate was applied across all age-groups, this being assumed to be a worst case for comparison with deterioration in non-COVID disease burden.

The details of assumptions involved in DALY calculations are provided in the supplementary file. Potential impact of reduced health service access in Uganda through the COVID-19 response was predicted for HIV/AIDS, malaria, and maternal mortality. HIV/AIDS predictions assumed an arbitrarily low (20%) loss to follow-up (no medication) for current infections extending for 6 months, with mortality returning to 1990 levels (essentially pre-ART). Reduced detection of new HIV infections and initiation of management is based on first quarter 2020 data.^25^

Excess malaria burden was estimated based on 6 months of incidence and mortality rate changes recently predicted by the WHO for three scenarios of minor, moderate, and major reductions in services (WHO scenario 1 [WS1]: no insecticide-treated net [ITN] campaigns, continuous ITN distributions reduced by 25%, WS4: no ITN campaigns, access to effective antimalarial treatment reduced by 25%, and WS9: no ITN campaigns, both continuous ITN distributions and access to effective antimalarial treatment reduced by 75%).^31^ Relative malaria mortality and incidence rates by age for Uganda were derived from the Institute of Health Metrics data,^32^ with the 2018 baseline mortality reported by the WHO.^18^

Maternal mortality was based on Uganda data from 2019 to 2020 and the data include only mortality, not persisting injury (see Results section).

### Ethical considerations.

The study used publicly available secondary aggregate-level data. No individual person-identifying information was used.

## RESULTS

### Trends in reported disease data.

As social-distancing policies were only instituted in Uganda in March 12, 2020, data accruing from the mandatory lockdown policy are limited, although prior knowledge of COVID-19 may have influenced prior health-seeking behavior.

#### HIV/AIDS.

**Figure 1.**
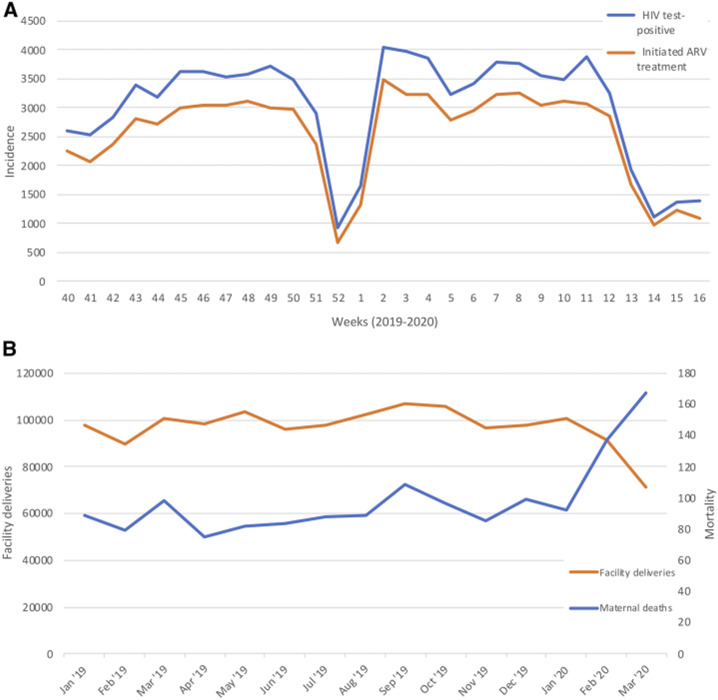
(**A**) HIV incidence and initiation of antiretroviral therapy in Uganda, week 40 (October 2019) to week 14 (mid-April) 2020.^25^ (**B**) Facility deliveries and maternal mortality, Uganda, 2019–2020.

New HIV case declined by 75% in the first 2 weeks of April, with a similar 75% reduction in the initiation of isoniazid-preventive therapy to prevent secondary tuberculosis.^25^ These dips were similar to a temporary decline over the 2019–2020 Christmas–New year period that subsequently recovered (Figure 1, Supplemental Table S2).

#### Malaria.

Malaria showed a reduction in case detection in the first quarter of 2020, consistent with a seasonal decline but more persistent and of greater magnitude than 2019 (1,206,606, 1,010,524, and 678,176 cases in January, February, and March 2020, respectively, versus 832,499, 666,493, and 722,370 in 2019). Admissions and inpatient deaths declined by similar proportions (Supplemental Table S3). This is difficult to interpret without a longer 2020 recording period, as it is consistent both with a larger seasonal decline in 2020 and with reduced health service and facility access (Supplemental Figure S1).

#### Maternal mortality.

A 29% (28,939) reduction in facility deliveries is recorded in the Ministry of Health Uganda data in March compared with January 2020, 28% less than the 12-month average for 2019. Over the same period, an 82% increase in maternal mortality was recorded (from 92 to 167 women), an increase of 87% over the 12-month 2019 average of 89.5 (Figure 1, Supplemental Table S4).

### Demography, predictions of DALYs lost, and mortality.

#### COVID-19.

**Figure 2.**
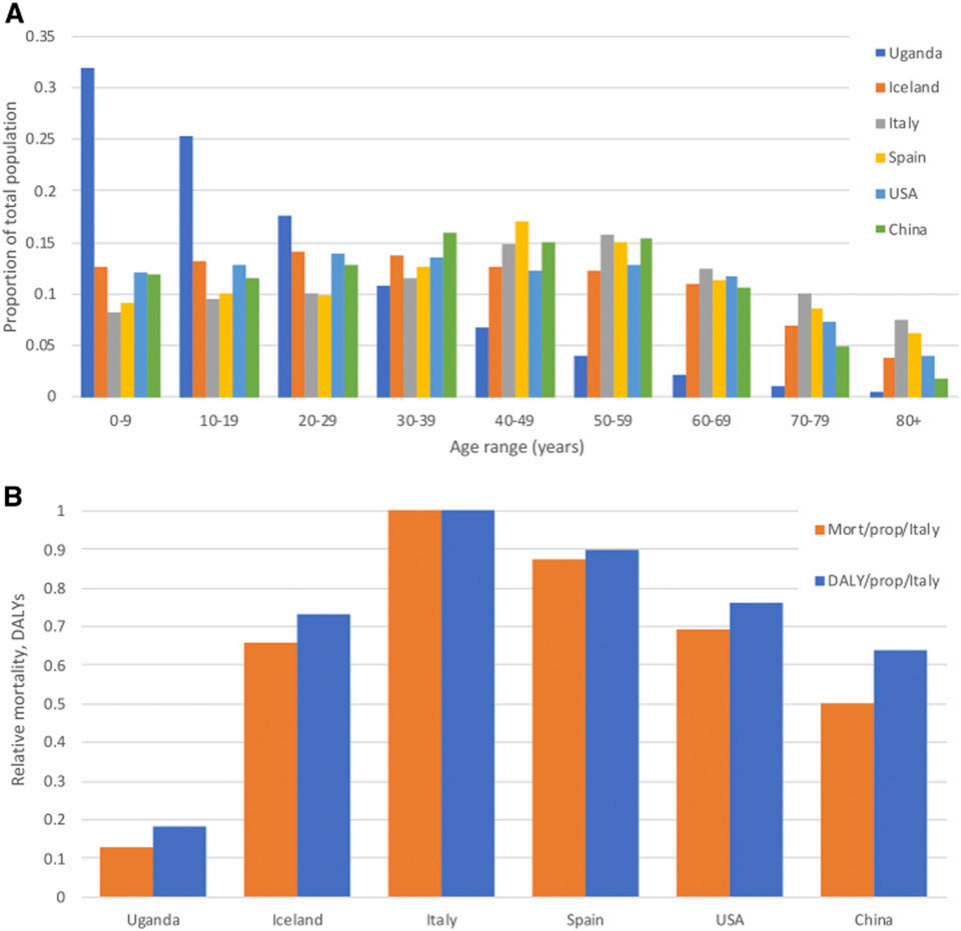
(**A**) Comparison of age profiles of Uganda with countries with COVID-19 outbreaks previously recorded and instituting varying degrees of physical distancing policies: China, the United States, Spain, Italy, and Iceland. (**B**) Relative mortality and burden of disability-adjusted life years from COVID-19 predicted by age distribution alone, assuming an equivalent infection rate per country, standardized against the burden predicted for Italy. Age-specific infection fatality rate is based on China.^33^

Data in 10-year age-groups for Uganda and comparator countries demonstrate a vastly different population age distribution (Figure 2). If the SARS-COV-2 infection rate is assumed to reach 20% of the total population in each country and the age-related mortality rate is based on that of China,^33^ a relatively lower disease burden is predicted for Uganda than all comparison countries, with mortality 0.13 and DALYs lost 0.19 that of Italy per head of population if equivalent transmission were to occur (Figure 2, Table 1). The bulk of the COVID-19 burden in Uganda is predicted to fall in late middle age, peaking in the 60- to 69-year age bracket (Table 2).

**Table 1:** Predicted mortality and DALYs lost for Uganda and comparator countries, based on age-related mortality from the China outbreak, and assuming a 20% detectable infection rate

	Uganda	Iceland	Italy	Spain	United States	China
Mortality	14,640	619	166,955	112,792	632,999	1,994,996
DALYs	405,100	13,109	3,180,727	2,215,642	13,307,928	48,406,233
Total population*	41,590,300	341,250	60,461,828	46,754,783	331,002,647	1,439,323,774
DALY/population	0.00974	0.0384147	0.052607189	0.047388566	0.040204898	0.033631233
Mortality/population	0.000352	0.0018144	0.002761321	0.002412408	0.001912368	0.001386065

DALYs = disability-adjusted life years.

* Population estimates obtained from the Uganda Bureau of Statistics and UN World Population Prospects.^24,30^

**Table 2:** DALYs lost in Uganda assuming a 20% infection rate

Age range	Age of death (years)	Number of deaths*	DALYs lost
0–9	5	42	3,696
10–19	15	147	11,355
20–29	25	452	30,320
30–39	35	755	43,169
40–49	45	892	42,180
50–59	55	2,028	76,028
60–69	65	3,293	91,733
70–79	75	3,965	73,836
80+	85	3,064	32,783
Total	–	14,640	405,100

DALYs = disability-adjusted life years.

* Based on mortality in China.^33^

#### HIV/AIDS.

Assuming a 20% loss to follow-up for 6 months among people living with HIV and a consequent return to 1990 mortality levels (no treatment scenario) for this population, and assuming an equal mortality across age-groups, 463,168 excess DALYs are predicted to be lost (Supplemental Table S5A). The burden from undiagnosed new HIV cases, based on the 55% decline in new ART initiation in April 2020 and using standard assigned HIV DALYs, predicts 41,757 excess cases failing to commence therapy over 6 months, with 12,151 DALYs lost (Supplemental Table S5B). Combined, this predicts 475,319 DALYs lost (Table 3)

**Table 3:** Comparison of predicted DALYs lost from COVID-19 if the national detectable infection rate reaches 20%*, and potential response vulnerabilities over 6 months, based on assumptions as listed

Cause	Basis of prediction	Excess DALYs lost	Notes
COVID-19	20% incidence	405,100	20% total detectable incidence nationally
HIV/AIDS	Missed new diagnoses and loss to follow-up†	475,319	Standard HIV DALYs. Missed new treatment initiation based on March 2020 vs. 2019 6-month average, and assumed 20% loss to follow-up reverting to 1990 HIV mortality rate
Malaria	WHO scenario 1‡	257,780	No ITN campaigns, continuous ITN distributions reduced by 25%
	WHO scenario 4‡	509,393	No ITN campaigns, access to effective antimalarial treatment reduced by 25%
	WHO scenario 9‡	2,427,769	No ITN campaigns, both continuous ITN distributions and access to effective antimalarial treatment reduced by 75%
Maternal mortality	March 2020 mortality rate†	31,343	March 2020 rate vs. 2019 6-month average

DALYs = disability-adjusted life years; ITN = insecticide-treated net.

* Mortality for detectable infection rate based on incidence recorded in China,^33^ and including only diagnosed and recorded infections.

† Uganda 2020 data. See Results.

‡ WHO.^18,31^

#### Malaria.

The Ugandan population is predicted to be significantly impacted if the WHO scenarios are realized, reflecting the low age at which most mortality occurs: mortality and total DALYs lost for WHO scenarios being 3,209 and 257,780 (WS1), 6,533 and 509,394 (WS4), and 31,046 and 2,427,769 (WS9), respectively (Table 3, Supplemental Table S6A and B).

#### Maternal mortality.

Based on the observed increase in maternal mortality in early March 2020 compared with the preceding 2019–2020 average, an excess 486 deaths are predicted for a 6-month period, incurring 31,343 DALYs lost (Supplemental Table S7). Non-fatal morbidity has not been taken into account.

Relative costs in DALYs lost and mortality for the modeled scenarios are summarized in Table 3.

## DISCUSSION

Based on population age structure alone, Uganda appears relatively strongly protected from severe COVID-19, with an equivalent sized outbreak imparting a far lower predicted COVID-19 burden per head of population than all comparator countries, less than a third that of China to less than a fifth that of Italy. Within Uganda, a very widespread COVID-19 outbreak (20% of population testing positive) is predicted to impart a lower burden than potential lockdown impacts in HIV/AIDs and malaria programs alone. Early data showing rising maternal mortality and reduced facility-based deliveries, reduced case finding for HIV/AIDS, and possibly malaria indicate deterioration in essential health service access associated with the COVID-19 pandemic and risk the reversal of hard-won gains in addressing fundamental vulnerabilities in women’s and children’s health.

A significant assumption here is that age-related mortality in Uganda would be similar to that in China. The effects of undernutrition, anemia, intestinal parasite infestation, and infectious disease such as tuberculosis and HIV are unknown. They are widely assumed to increase susceptibility, as may chronic lung conditions from indoor and outdoor air pollution. However, major risk factors recorded for COVID-19, such as obesity, diabetes, hypertension, and other cardiovascular diseases,^34,35^ are generally less prevalent in sub-Saharan populations.^36^ In Uganda, a recent national survey reported the prevalence of diabetes at 1.4%.^36^ It is difficult to predict whether the population at a given age will be more or less susceptible. Lower access to intensive care, and particularly to oxygen, may reduce recovery rates for severe cases in Uganda and more broadly in this region in DALYs lost and mortality.^37,38^ We also make no adjustments for the impact of age structure on underlying transmission, but compare the age-based predicted mortality for a hypothetical equivalent transmission uniformly across each age-group.

To address these uncertainties, this article has taken a conservative approach to comparing COVID-19 impact with that of lockdown-related program deterioration, assuming a very high transmission of COVID-19 (a 20% detectable infection rate) across Uganda. As “detectable infection” here is based on early China data when testing was scaling up, and subsequent data suggest that asymptomatic SARS-CoV-2 infection is relatively frequent,^39^ the COVID-19 burden predicted here would reflect a population infection rate considerably higher than 20%. It is noted that countries with major established outbreaks have far lower levels of detectable infection overall.^23^ The predictions for COVID-19 disease burden used here in comparison to other disease burdens are intended as a worst-case scenario for COVID-19, and probably greatly overestimate the COVID-19 impact.

The fragility of the Uganda HIV/AIDS burden is reflected in the high predicted DALYs lost with moderate reductions in health service access, similar to that predicted with a widespread SARS-COV-2 transmission producing 20% detectable infection across the population. This prediction here may be conservative regarding long-term burden as HIV/AIDS high infant mortality might be expected through increased maternal–infant transmission, whereas adult-to-adult transmission will also increase if access to ART and condoms is reduced. The reduction in HIV/AIDS case finding is early and may not be sustained, whereas impact on mortality will also take time to accrue. We have used a lower rate of loss to follow-up than shown in the PEPFAR data (20%) to account for this, but this is an area where further data are needed. Later data from June 7 (week 23) show both HIV case finding and treatment initiation remaining 37% less than the 26-week average end of March, suggesting the estimates are indeed conservative.^40^

The use of DALYs lost, rather than reliance solely on incidence and mortality, seems essential to understand COVID-19 burden. With the burden of COVID-19 heavily concentrated in older age-groups,^33,35^ each death imparts a far lower burden than is commonly accrued from HIV/AIDs, malaria, or a maternal death. Maternal and HIV deaths not only remove many years of a parent’s life but also frequently leave children orphaned, with further resultant costs. The predictions in this article, although based on fairly modest assumptions of program deterioration from only three disease areas, indicate a considerable risk of collateral damage to other disease programs compared with the COVID-19 burden from an extensive outbreak predicted here (Table 3). Increases in infant malnutrition and stunting through economic impact of the response, not modeled here, will have impact for years to come. The World Food Program predicts up to 130 million additional people will face acute food insecurity globally, a large proportion in sub-Saharan Africa.^41^ The impact of interruption of vaccination programs will increase the longer health service interruptions persist, with outbreaks including measles in neighboring Democratic Republic of Congo as a growing threat.^42^ An expected 2.1–5.1% reduction in gross domestic product across sub-Saharan Africa will impede the ability of health systems and populations to recover,^43^ whereas reduction in support from donor countries is a risk as they deal with economic downturn at home.

Understanding the extent of spread in the community through better testing will be important to understand whether the relatively low COVID-19 numbers recorded in African countries to date are due to late introduction, are an artifact of low testing, or are due to the low rate of severe presentations predicted here. The consequences of getting the equation wrong are dire. Ramping up testing capacity, prioritizing the building of an evidence base on COVID-19 epidemiology in African populations, is urgent if the risks raised in this study are to be minimized.

A broader modeling approach to COVID-19 is needed, incorporating accepted measures of disease burden rather than solely mortality, and including comparisons for collateral damage due to the public health response based on emerging health system data. Sentinels for deterioration such as facility-based delivery, maternal mortality, and case finding for HIV and tuberculosis should provide bases for this modeling. Uganda’s relatively strong surveillance and monitoring networks provide a strong basis for this across a range of diseases.

## CONCLUSION

Our predictions strongly suggest that on the basis of DALYs lost, the impact of the COVID-19 public health response on non–COVID-19 diseases could outweigh the direct impact of an extensive COVID-19 outbreak. Although public health responses to COVID-19 should be prioritized, prior morbidities should not be de-prioritized. COVID-19 is predicted here to cause considerably less direct impact than predicted elsewhere, and the response in Uganda, and by extension elsewhere where population age structure is similar, should be strongly tailored to local context.

## Supplemental figure and tables

Supplemental materials
