# Supplementary figures and images for: Predicting the Impact of COVID-19 and the Potential Impact of the Public Health Response on Disease Burden in Uganda
Source: Am J Trop Med Hyg. 2020 Jul 23;103(3):1191–7. doi: 10.4269/ajtmh.20-0546 (PMC7470592; doi:10.4269/ajtmh.20-0546)

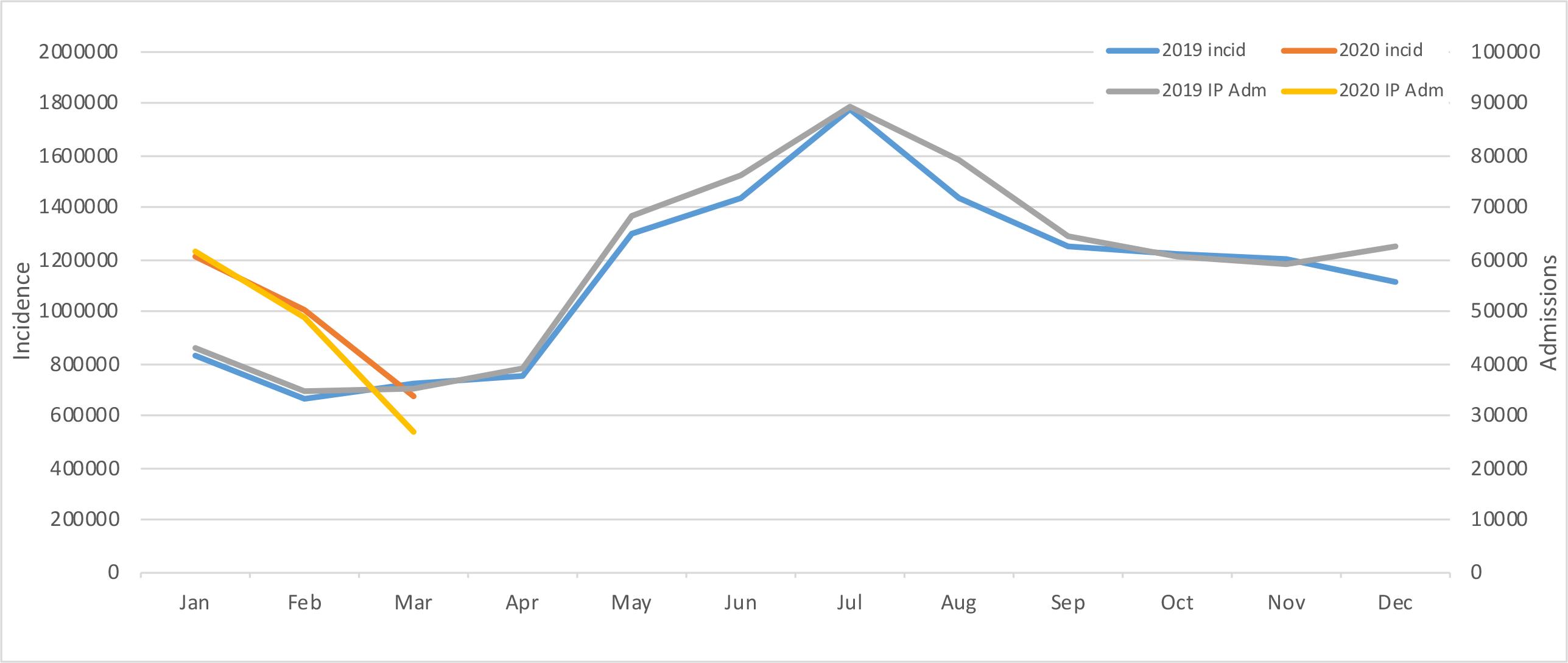

Supplement: Supplementary file 1 [file tpmd200546.SF1.tif]
